# Life-Threatening Overt Small Bowel Bleeding From Jejunal Crohn’s Disease

**DOI:** 10.7759/cureus.97950

**Published:** 2025-11-27

**Authors:** Carlos Elizondo Alatorre, Julio Valencia, Gabriela Rubianes, Pushkar Kumar, Jennifer Harley

**Affiliations:** 1 Internal Medicine, NYC Health + Hospitals/Metropolitan, New York, USA; 2 Gastroenterology and Hepatology, NYC Health + Hospitals/Metropolitan, New York, USA

**Keywords:** crohns, gastrointestinal bleeding, ibd, jejunal crohns disease, small bowel bleeding

## Abstract

Bleeding from the small bowel is relatively uncommon, and when suspected, its diagnosis can be challenging. It often requires additional, and sometimes multiple, endoscopic techniques to accurately identify the source. Jejunal Crohn’s disease (CD) is rare and carries a higher risk of complications compared to other forms of CD. Severe anemia (hemoglobin <10) at the time of CD diagnosis is also unusual. We present a case of a 23-year-old female with obscure gastrointestinal bleeding and life-threatening anemia, who was ultimately diagnosed with jejunal Crohn’s disease. This case highlights the intersection of three rare findings: small bowel bleeding, jejunal CD, and severe anemia at presentation.

## Introduction

Small intestinal bleeding is uncommon, accounting for approximately 5% to 10% of all patients with gastrointestinal bleeding [1]. Prior to the early 2000s, it was difficult to characterize and detect using standard bidirectional endoscopy. However, with the advent of video capsule endoscopy (VCE) and device-assisted enteroscopy, small bowel bleeding is now better visualized and is no longer routinely classified under the umbrella of obscure gastrointestinal bleeding [1].

Crohn’s disease (CD) is a chronic idiopathic inflammatory bowel disease (IBD) characterized by skip lesions and transmural inflammation, potentially affecting the entire gastrointestinal tract [2]. CD can present with different symptoms, including abdominal pain, diarrhea, weight loss, and fever, and up to 80% of patients require hospitalization at some point during their disease [3,4]. Severe anemia (Hgb <10) at the time of diagnosis has been reported in only 6% of patients with CD, as shown in a large retrospective cross-sectional study [5]. Ileocolonoscopy with biopsies is key for CD diagnosis [4].

Half of CD cases affect the terminal ileum and colon; however, upper gastrointestinal Crohn’s disease (UGI-CD) affects 13% of cases [6]. Jejunal involvement in CD is an independent risk factor for stricturing and for having multiple surgeries [4,7,8]; however, there is limited data on its true prevalence. One study found jejunal lesions in 56% of patients with CD, with isolated jejunal involvement in only 17% [9]. A Korean cohort reported jejunal disease at diagnosis in 14.1% of patients [10].

Diagnosing jejunal CD is challenging, as traditional endoscopy and colonoscopy cannot reach the entire jejunum [11]. We present a case of a 23-year-old female with recurrent episodes of melena leading to severe anemia, ultimately diagnosed with jejunal Crohn’s disease.

## Case presentation

A 23-year-old woman with a 5-year history of chronic iron-deficiency anemia was first hospitalized three months prior after presenting with a 2-day history of dark stools, headache, and laboratory evidence of severe anemia (hemoglobin 4 g/dL; reference 12-16 g/dL) and a positive digital rectal examination for melena. During that admission, both upper endoscopy and colonoscopy were unremarkable. She received blood transfusions, her hemoglobin stabilized, bleeding resolved, and she was discharged with outpatient follow-up.

**Figure 1:**
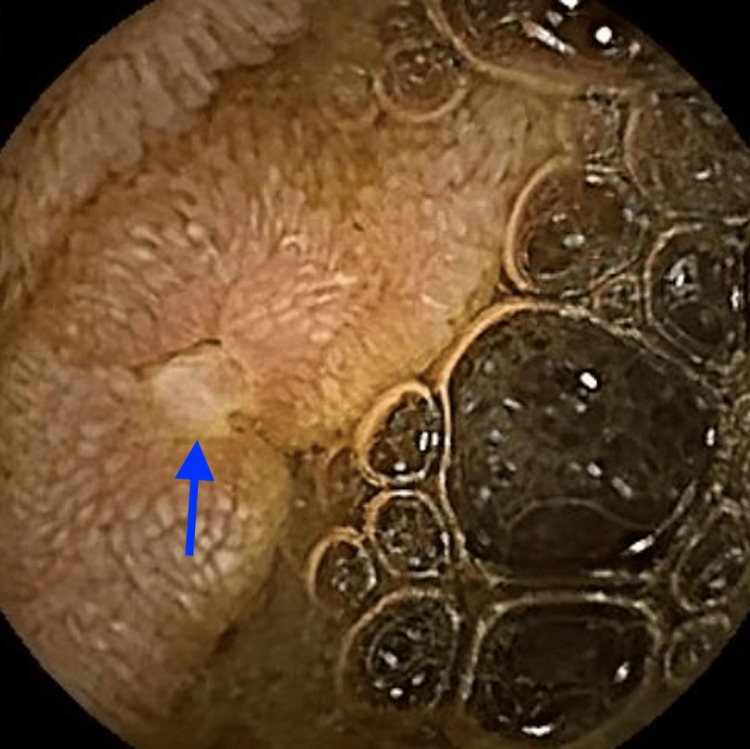
Video capsule endoscopy revealing a jejunal ulcer (indicated by the arrow)

**Figure 2:**
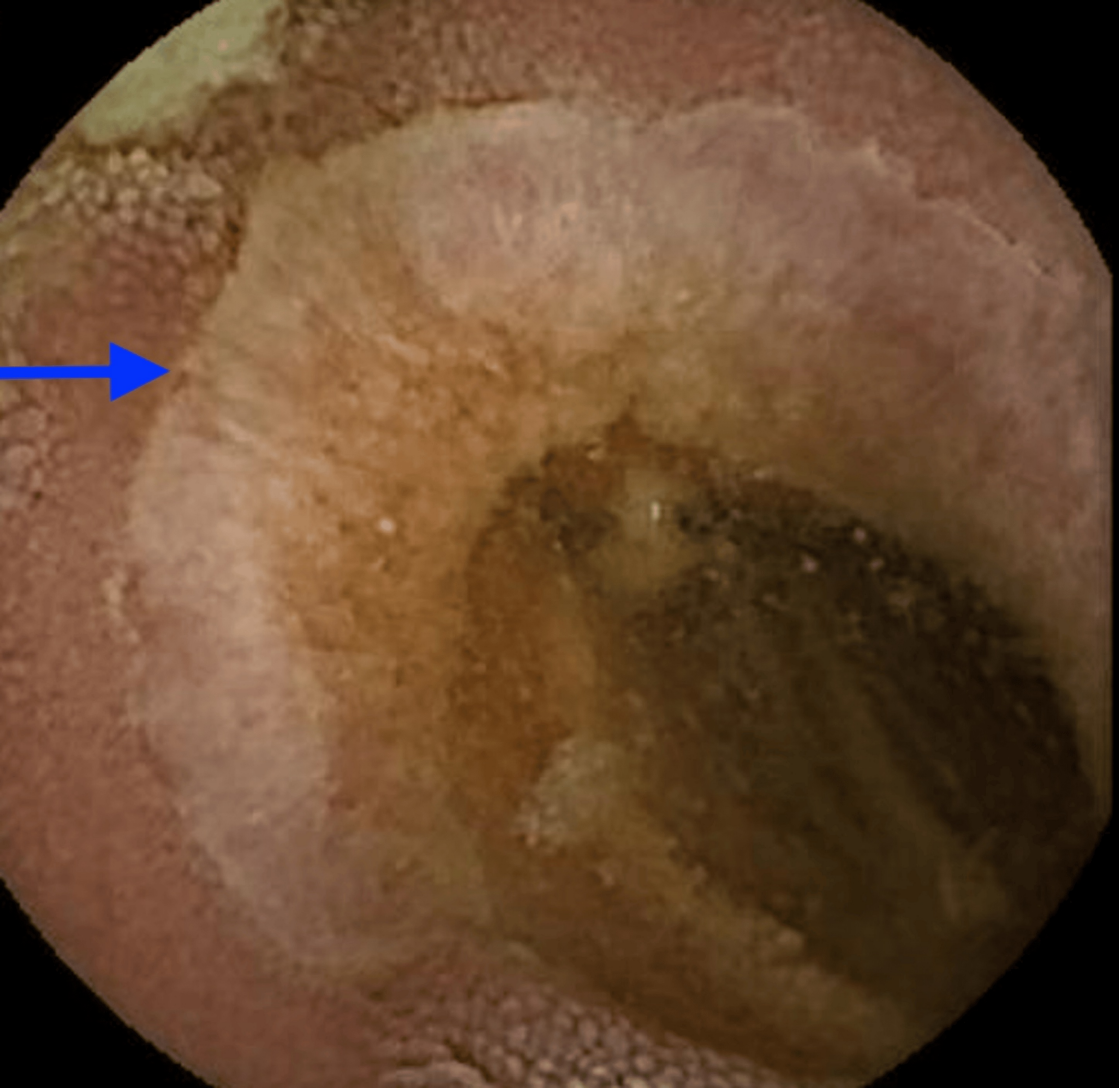
Video capsule endoscopy revealing a jejunal ulcer (indicated by the arrow)

**Figure 3:**
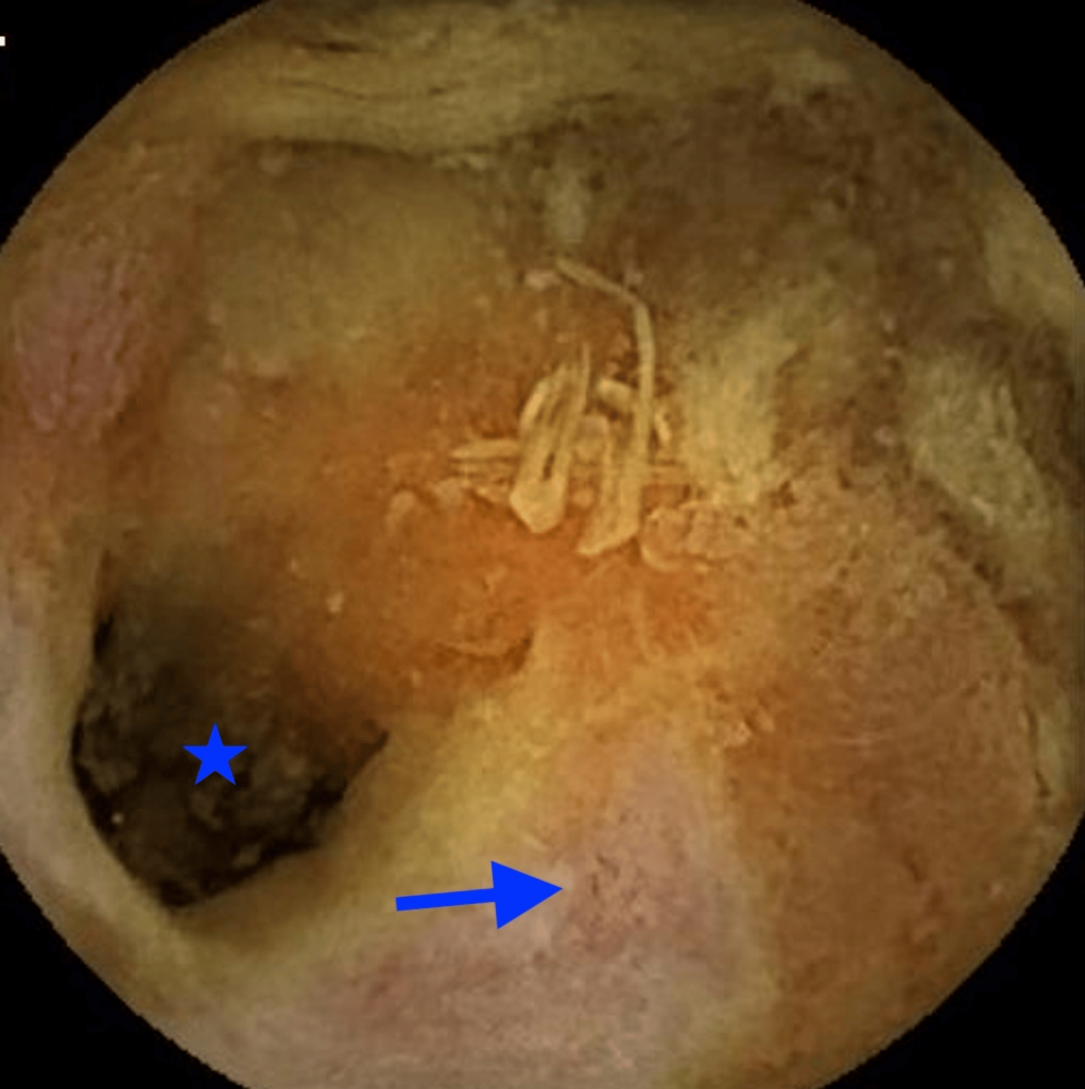
Video capsule endoscopy demonstrating an ulcer (indicated by the arrow) and a stricture (marked by the star) in the jejunum

As an outpatient, she underwent VCE, which demonstrated multiple ulcerations (indicated by arrows) and strictures (marked by stars) in the jejunum (Figures 1-3). Laboratory tests showed fecal calprotectin 1,550 µg/g (reference <49 µg/g) and C-reactive protein 18.6 mg/L (reference <5.1 mg/L), raising concern for CD. A push enteroscopy reaching the proximal jejunum did not reveal any bleeding source. CT enterography was ordered but had not yet been performed when she again developed symptoms.

Three months later, she presented to the emergency department with a three-day history of black tarry stools and a two-day history of severe pulsating headache. She denied hematemesis or hematochezia.

On arrival, she appeared pale but alert. Vital signs were blood pressure 108/65 mmHg, heart rate 96 bpm, temperature 36.7 °C, and oxygen saturation 98% on room air. Laboratory testing revealed hemoglobin 4 g/dL (reference 12 g/dL -16 g/dL) and a positive rectal examination for melena. She was transfused with packed red blood cells and admitted for further evaluation.

Repeat upper endoscopy and push enteroscopy were unremarkable. The patient was subsequently transferred to a tertiary center, where double-balloon enteroscopy (DBE) revealed multiple deep ulcers in the jejunum and proximal ileum. Biopsies showed severe active enteritis with mucosal erosion and epithelioid granulomas, confirming the diagnosis of Crohn’s disease. DBE and histopathology images were unavailable, as the procedures were performed at an outside facility.

She was started on oral budesonide and was later transitioned to risankizumab for maintenance therapy.

## Discussion

Small bowel bleeding should be considered in patients with gastrointestinal bleeding after a normal upper endoscopy and colonoscopy. In individuals under 40 years old, one of the most common causes of small bowel bleeding is inflammatory bowel disease, particularly CD [1]. CD is known for its potential to affect the entire gastrointestinal tract, from the mouth to the anus [2]. The global prevalence of CD has been rising, reaching approximately 224.2 per 100,000 people in 2019 [12].

Half of patients with CD have involvement of the terminal ileum and colon [2]. The presence of UGI-CD is often underestimated; a meta-analysis by Chin et al. reported that UGI-CD is present in 13% of patients with CD, including disease involving the esophagus, stomach, duodenum, jejunum, and proximal ileum [6]. However, it remains uncertain whether jejunal disease should be considered a different entity or belong to the classification of UGI-CD [13]. Forty percent to 55% of patients with CD require a major abdominal surgery within 10 years, and their mortality is elevated with a standardized mortality ratio of 1.4 compared to the general population [4]. Specifically, jejunal disease has been associated with an increased risk for strictures and abdominal surgery compared to other CD locations [6-8], underscoring the importance of early diagnosis to prevent complications and/or surgical intervention. Our patient’s presentation aligns with these observations, as the presence of jejunal ulcerations and strictures on VCE reflects an aggressive disease phenotype with high potential for complications. Most cases of small intestinal bleeding are undramatic [1]; in contrast, our patient presented with life-threatening anemia (Hgb 4 g/dL), which contrasts with the typical presentation described in prior reports.

Colonoscopy’s reach is limited to 5 to 10 cm of the terminal ileum, and although push enteroscopy can reach up to 120 cm distal to the ligament of Treitz, it still fails to visualize the deep small bowel [11]. Patients with CD may have inflammation more proximally than the terminal ileum, which is out of reach of the colonoscope [14]. A retrospective cohort study by Solitano et al. demonstrated that colonoscopy missed detection of active CD in 22.3% of cases due to more proximal disease location [14]. Our case aligns with these findings, as both conventional upper and lower endoscopy failed to detect pathology. Technological advances have improved small bowel evaluation through VCE or DBE, and cross-sectional imaging such as computed tomography enterography or magnetic resonance enterography [15]. Our case reinforces these studies by highlighting the diagnostic value of VCE and DBE in identifying jejunal CD after nondiagnostic conventional endoscopy.

## Conclusions

In conclusion, although rare, small bowel bleeding can present with life-threatening anemia, emphasizing the importance of advanced diagnostic tools for accurate evaluation. In patients under 40 presenting with overt small bowel bleeding, jejunal Crohn's disease (CD) should be considered, as it is associated with a higher risk of complications, which often require major abdominal surgeries. Early and accurate diagnosis is crucial not only in the acute phase but also to prevent long-term morbidity. Furthermore, a clear definition of where jejunal CD fits within the current classification is essential.
